# The Many Faces of the Angry Peritoneum

**DOI:** 10.3390/diagnostics15091163

**Published:** 2025-05-03

**Authors:** Maria Chiara Ambrosetti, Matilde Bariani, Giulia Angela Zamboni, Riccardo Valletta, Matteo Bonatti

**Affiliations:** 1 Radiology Unit, Department of Pathology and Diagnostics, Azienda Ospedaliera Universitaria Integrata of Verona, 37126 Verona, Italy; matilde.bariani@aovr.veneto.it; 2 Institute of Radiology, Department of Diagnostics and Public Health, Policlinico GB Rossi, University of Verona, 37134 Verona, Italy; gzamboni@hotmail.com; 3 Department of Radiology, Hospital of Bolzano (SABES-ASDAA), Teaching Hospital of Paracelsus Medical University (PMU), 39100 Bolzano, Italy; riccardo.valletta@gmail.com (R.V.); matteobonatti@hotmail.com (M.B.)

**Keywords:** peritoneum, peritoneal carcinomatosis, disease pathways

## Abstract

The peritoneum is a thin membrane that lines the abdominal cavity and covers the abdominal organs. It serves as a conduit for the spread of various pathological processes, including gas and fluid collections, inflammation, infections, and neoplastic conditions. Peritoneal carcinomatosis is the most common and well-known pathology involving the peritoneum, typically resulting from the dissemination of gastrointestinal and pelvic malignancies. However, numerous benign and malignant peritoneal diseases can mimic the imaging appearance of peritoneal carcinomatosis. The aim of this review is to revisit the anatomy of peritoneal compartments and elucidate the patterns of peritoneal disease spread. Emphasis is placed on identifying the distinctive imaging features of both neoplastic and non-neoplastic peritoneal diseases that differ from peritoneal carcinomatosis.

## 1. Introduction

### 1.1. The Peritoneum and the Peritoneal Spaces

The peritoneum is a thin, transparent membrane that consists of two layers: the parietal peritoneum, which lines the abdominal cavity, and the visceral peritoneum, which covers the abdominal organs. A small amount of serous fluid, which acts as a lubricant, reduces friction during organ movements and separates these two peritoneal layers [1,2,3]. The peritoneal cavity is the space between the two peritoneal layers, which is enclosed in men and communicates with the outside through the fallopian tubes in women. This cavity is further divided into the greater and lesser sacs, which communicate with one another through the foramen of Winslow. The greater sac extends across most of the peritoneal cavity, whereas the lesser sac is located posterior to the stomach and anterior to the pancreas [3,4,5]. The peritoneal folds, including the mesenteries, omenta, and ligaments, provide support and connections between the organs. Specifically, the mesenteries support the intestines, while the great omentum is anchored at the stomach level. The lesser omentum connects the stomach to the liver, providing a pathway for blood vessels, lymphatics, and nerves [3,5,6,7].

### 1.2. Peritoneal Disease Extension Pathways

A variety of diseases can spread within the peritoneum and peritoneal spaces, including gas and fluid collections, inflammation, infections, and neoplastic processes. Several mechanisms contribute to this spread: direct extension spread along the ligaments, the perineural and perivascular pathways, as well as hematogenous and lymphatic diffusion [8].

The spread of peritoneal diseases is influenced by the peritoneal folds, ligaments, and the flow pattern of peritoneal fluid that moves downward due to gravity and upward during inspiration, driven by subdiaphragmatic negative pressure [1]. The fluid can easily spread bidirectionally within the right paracolic gutter, while flow in the left paracolic gutter is restricted by the phreno-colic ligament. Consequently, intraperitoneal diseases are more frequently observed on the right side of the abdomen than on the left [8]. Sites of fluid stasis are also more likely to serve as locations for disease seeding. These include the rectovesical and rectouterine pouches, the ileocolic region, the right paracolic gutter, the right subdiaphragmatic space, and the superior border of the sigmoid mesocolon [9].

Peritoneal ligaments and structures are typically involved in various diseases, depending on their location and the organs they connect. The transverse mesocolon, for example, is closely associated with the pancreas, stomach, colon, and duodenum and serves as a connection between the peritoneum and retroperitoneum, allowing disease to spread between these structures [8,9,10].

Perineural spread may be difficult to detect on imaging, but it can sometimes be identified as neoplastic tissue spreading along the blood vessels [11].

Lymphatic spread along the diaphragmatic surface is often observed in non-Hodgkin lymphomas, as well as ovarian and gastrointestinal cancers [8].

## 2. Method/Search Strategy

We searched our departmental databases for cases involving the peritoneum which have been studied at our radiology units in Verona and Bolzano over the past three years. We collected a selection of both common and rare manifestations of peritoneal involvement. Our focus was on identifying neoplastic and non-neoplastic conditions that exhibit imaging features mimicking peritoneal carcinomatosis. At the same time, we placed particular emphasis on peritoneal diseases distinct from peritoneal carcinomatosis, aiming to highlight the specific imaging findings of rarer conditions with which radiologists should be familiar. For each pathology, we conducted a literature review to outline the key clinical features and current management strategies. This approach was intended to support radiologists in achieving accurate diagnoses and to assist clinicians in selecting appropriate therapeutic options for patient care. To enhance our understanding of the imaging characteristics of these diseases, we first reviewed the anatomy of the peritoneum, the pathophysiological mechanisms involved, and the various routes of disease spread. For clarity, we categorized the collected conditions into four groups: malignant neoplastic, benign neoplastic, inflammatory, and traumatic.

The aim of our work was to compile a comprehensive overview of both common and rare peritoneal abnormalities that may pose a differential diagnostic challenge with peritoneal carcinomatosis, in order to assist radiologists—particularly those with less experience—in making accurate differential diagnoses.

## 3. Peritoneal Diseases

### 3.1. Neoplastic Malignant

#### 3.1.1. Peritoneal Metastatic Disease

Peritoneal metastatic disease is the most common form of peritoneal neoplasm. It can originate from epithelial cells (peritoneal carcinomatosis), mesenchymal cells (peritoneal sarcomatosis), or lymphoid cells (peritoneal lymphomatosis).

Peritoneal carcinomatosis (PC) refers to the dissemination of neoplastic epithelial cells into the peritoneal cavity [12,13]. It is most associated with gastrointestinal and ovarian neoplasms. Only 10% of cases originate from extra-abdominal neoplasms, with the most frequent sources being breast cancer (41%), lung cancer (21%), and melanoma (9%) [14].

This condition is associated with a poor prognosis, with an average survival of approximately 6 months from the initial diagnosis.

**Figure 1:**
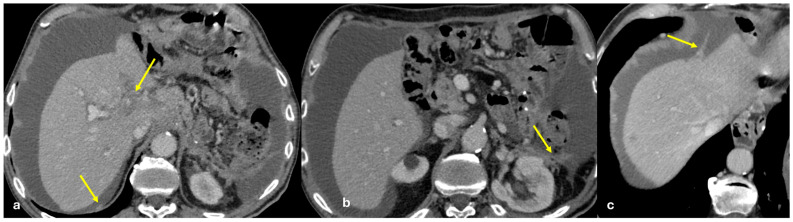
PC from gastric adenocarcinoma. An old man who underwent gastrectomy for gastric adenocarcinoma 2 years before. At follow up, axial CECT shows subtle soft tissue mass occupying the periportal space with vessel occlusion ((**a**), arrow), macro-nodular thickening of the posterior parietal peritoneum ((**b**), arrow), and micro-nodular thickening of the falciform ligament and posterior diaphragmatic peritoneal reflection ((**c**), arrow).

**Figure 2:**
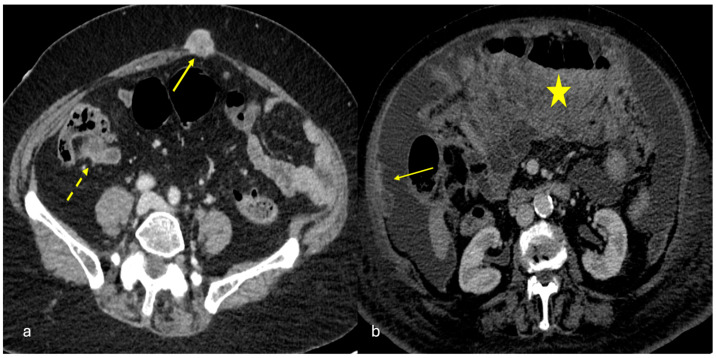
(**a**) PC from sigmoid carcinoma. An old woman. Follow-up CECT shows a macro-nodular implant located on the anterior abdominal wall (arrow) and a subtle deposit of the visceral peritoneum lining the last ileal loop (theca pattern, dashed arrow). (**b**) (Different case to (**a**)) PC from carcinoma of the colon. A middle-aged man. Follow-up CECT shows massive thickening of the omentum called an “omental cake” (star). Diffuse thickening of the peritoneal layer is also visible (arrow).

**Figure 3:**
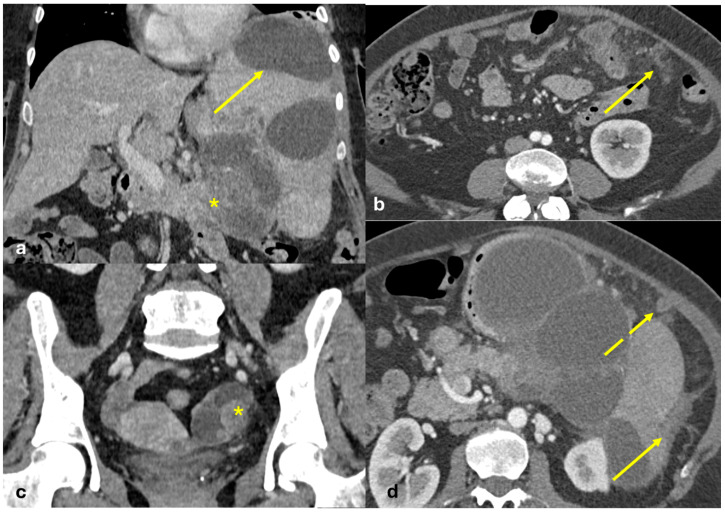
PC from pancreatic adenocarcinoma. An old woman with unresectable adenocarcinoma of the body–tail of the pancreas. CECT shows the primitive lesion ((**a**), coronal plane, asterisk) and large subcapsular deposits with splenic invasion along the lower surface of the left diaphragm ((**a**), arrow), micro-nodular omental deposits ((**b**) arrow and (**d**) dashed arrow), deposits along the left paracolic gutter ((**d**), arrow), and secondary lesions to both the ovaries ((**c**), asterisk).

**Figure 4:**
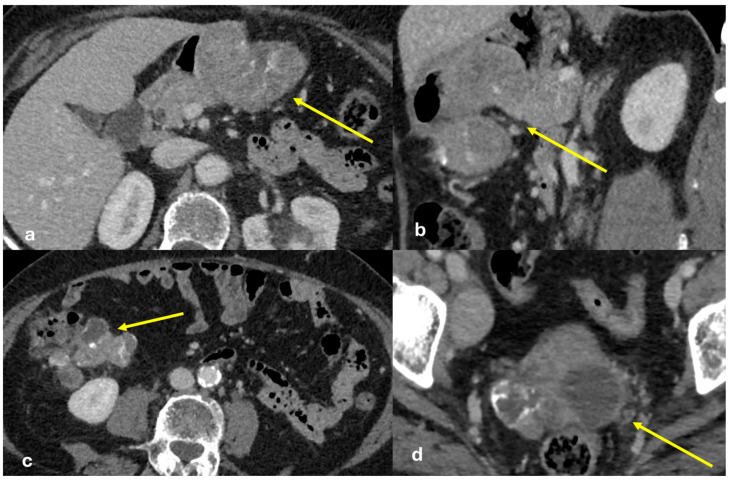
PC from ovary carcinoma. CECT of an old woman with large heterogeneus mass of the ovary (mass-like) with psammoma body ((**d**), arrow) and large confluent solid implants forming mass-like PC. Note the large nodules which are not cleavable from the stomach and duodenum ((**a**), axial, arrow, and (**b**), sagittal plane, arrow) and from the ascending colon ((**c**), axial plane, arrow).

Several patterns of peritoneal involvement have been described in the literature:

- Micro-nodular pattern: micro-nodules with a diameter ≤ 5 mm (Figure 1);
- Nodular pattern: nodules with a diameter > 5 mm (Figure 2a);
- “Omental cake”: nodular thickening of the omentum (Figure 2b);
- Plaque pattern: confluent nodular plaques, typically involving the lower surface of the right diaphragm (Figure 3);
- Mass-like pattern: confluence of nodules forming a large mass (several centimeters) (Figure 3 and Figure 4);
- Theca pattern: thickening of the visceral peritoneum lining the small bowel loops (Figure 2a) [15,16,17,18,19].

Often, these patterns coexist (Figure 1, Figure 2, Figure 3 and Figure 4). Additionally, other less common conditions can mimic peritoneal carcinomatosis and present with distinct patterns on imaging, as discussed in the following sections.

#### 3.1.2. Peritoneal Mesothelioma

Peritoneal mesothelioma is a rare malignant peritoneal neoplasm with symptoms that can vary widely depending on the tumor’s aggressiveness. More aggressive subtypes may lead to rapidly progressive symptoms, often related to mass effects, such as bowel obstruction. CT is the first imaging modality used to assess the overall tumor burden and the surgical resectability. MRI, especially with DWI, has better sensitivity for detecting smaller tumors.

**Figure 5:**
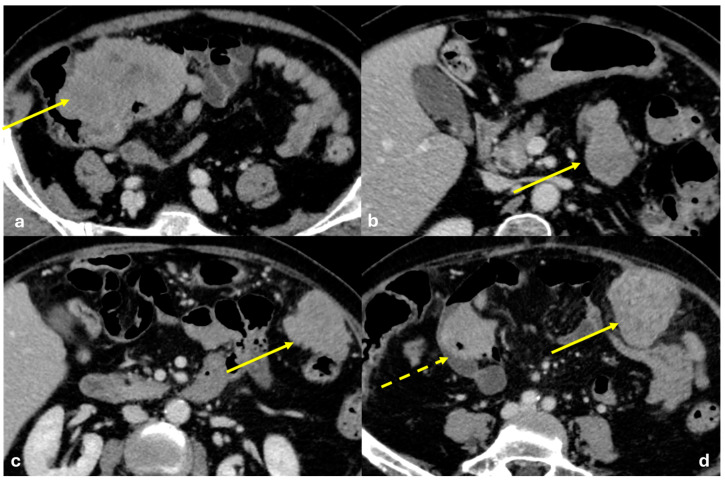
Peritoneal mesothelioma. An old man with abdominal pain. CECT on the axial plane reveals a large solid heterogeneous mass not cleavable to the cecum ((**a**), arrow) located in the mesentery ((**b**), arrow) along the large omentum ((**c**,**d**) arrows) and along the serosal surface of the small bowel ((**d**), dashed arrow).

Imaging findings can differ based on the morphological subtype of peritoneal mesothelioma, which includes wet-type, dry-type, and mixed-type. In the wet-type, ascites is predominant, with associated peritoneal thickening or plaques. In the dry-type, solid masses are usually detected without ascites (Figure 5). The mixed-type features both ascites and solid masses equally [20]. Peritoneal masses or plaques are typically hypodense, occasionally occurring with calcifications. When localized, peritoneal mesothelioma can present as a heterogeneous solid mass invading adjacent viscera. The most common histological subtype is epithelioid mesothelioma, which typically presents as peritoneal thickening, nodules, and ascites. Sarcomatoid mesothelioma, the most aggressive subtype, often manifests as a single or as multiple large peritoneal masses. Ascites is more commonly observed in the epithelioid subtype than in the sarcomatoid subtype [21]. As the disease progresses, small nodules merge into larger masses that infiltrate the mesentery and encase the solid organs and bowel loops. Initially, mesenteric thickening may cause small bowel kinking, with distortion being a common finding. As the tumor advances, mesenteric disease extends, leading to segmental bowel obstruction. The tumor can also invade the celiac, periportal, and epiphrenic regions, as well as pelvic sidewalls, retroperitoneal lymph nodes, and, in some cases, the sacrum [22]. Differential diagnoses include peritoneal carcinomatosis, primary peritoneal serous carcinoma, tuberculosis, and omental infarction.

#### 3.1.3. Peritoneal Localizations of GIST–Peritoneal Sarcomatosis

Gastrointestinal stromal tumors (GISTs) are the most common soft tissue sarcomas of the gastrointestinal tract. They originate from the interstitial cells of Cajal within the muscularis propria and most commonly arise in the stomach (60%), followed by the small intestine (30%), duodenum (5%), colon (4%), and esophagus/appendix (1%). Since GISTs develop from the myenteric plexus, they typically present as submucosal tumors with intramural growth beneath the mucosa. Their imaging characteristics vary depending on their location, growth pattern (endophytic, intramural, or exophytic), and enhancement pattern (hypervascular, intermediate, or delayed enhancement) [23].

Necrosis, calcification, ulceration, drainage veins, and regional/distant metastasis can be present. Small GISTs (<5 cm) tend to be round and homogeneous tumors, while large GISTs (>5 cm) tend to be lobulated and heterogeneous [24]. GISTs primarily metastasize to the liver (65%) and peritoneum (21–45%). The peritoneal dissemination of GISTs is termed peritoneal sarcomatosis, analogous to the peritoneal carcinomatosis seen in adenocarcinomas [25].

**Figure 6:**
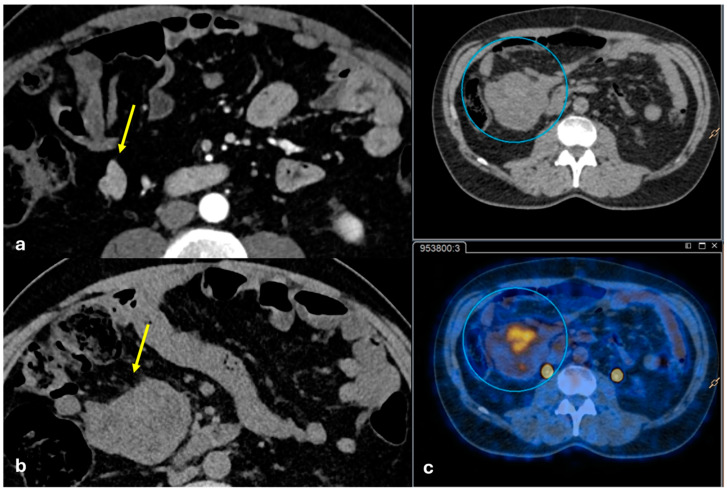
Peritoneal sarcomatosis. A middle-aged man. CECT was made during follow-up after GIST removal. In the first image, ((**a**), arrow) a hypervascular solid nodule is depicted in the mesentery. After 6 months, the same nodule became larger (CT (**b**), arrow) and showed FDG uptake at PET-CT (**c**).

Peritoneal implants from sarcomas are usually spherical, associated with minimal ascites, and rarely cause bowel obstruction or hydronephrosis, while carcinomatosis implants tend to be flat or ovoid and conform to the adjacent structures (Figure 6).

The peritoneal tumor burden in GISTs usually consists of large discrete masses, often being necrotic and with heterogeneous enhancement. Diffuse hypervascular omental and peritoneal caking are rare. Complications can include enteric fistulization due to tumoral necrosis or hemoperitoneum from gastrointestinal bleeding due to hypervascularity.

#### 3.1.4. Angiomyofibroblastoma

Angiomyofibroblastomas are extremely rare mesenchymal tumors. They are usually benign neoplasms, but recurrence and sarcomatous transformation have been reported.

They typically arise in women between 20 and 50 years of age and frequently involve the lower genital tract. They appear as a well-defined soft tissue mass, resembling Bartholin’s cysts when involving the lower genital tract [26,27]. Less frequently, they develop in the pelvis or peritoneal cavity, reaching massive sizes before diagnosis [28]. They consist of two components: stromal cells and prominent blood vessels [29].

Differential diagnosis may be challenging between angiomyofibroblastomas and other mesenchymal neoplasms, in particular aggressive angiomyxoma, but the latter has an infiltrative growth pattern generally not found in angiomyofibroblastomas [27].

**Figure 7:**
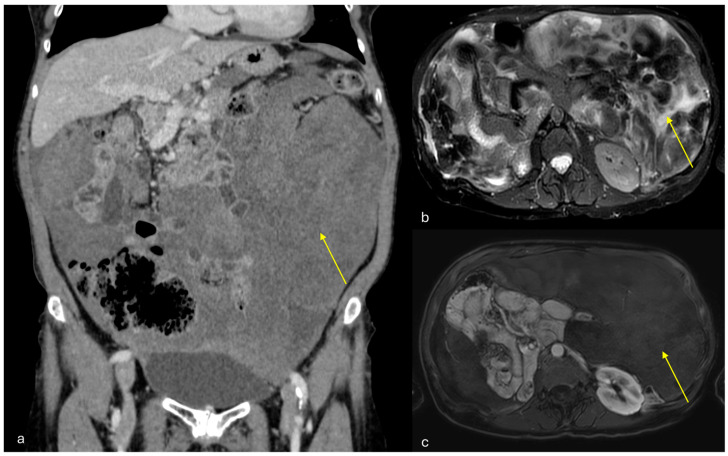
Angiomyofibroblastoma. A middle-aged woman. CT and MRI show a voluminous non-infiltrative peritoneal and pelvic lesion encasing the small bowel loops ((**a**): coronal portal venous phase CT; (**b**): axial T2-weighted MRI; (**c**): axial venous phase MRI. Indicated by arrows).

On ultrasound they appear as heterogeneous masses in superficial regions. On MRI, due to their fibrous content, they appear isointense to muscle on T1-weighted images and hypointense on T2-weighted images (Figure 7). After contrast administration, they demonstrate strong enhancement due to their prominent vascularity [27,28,29,30].

#### 3.1.5. Peritoneal Lymphomatosis

Extranodal lymphoma involvement occurs in approximately 40% of cases and has been described in almost every organ and tissue, being more common in non-Hodgkin’s lymphoma. Primary extranodal disease is less common than the secondary involvement of extranodal tissues as part of generalized disease. Peritoneal lymphomatosis is defined as the intraperitoneal spread of lymphoma and is a very rare clinical presentation for lymphoma. Omental involvement is uncommon because the omentum lacks lymphoid elements [31].

**Figure 8:**
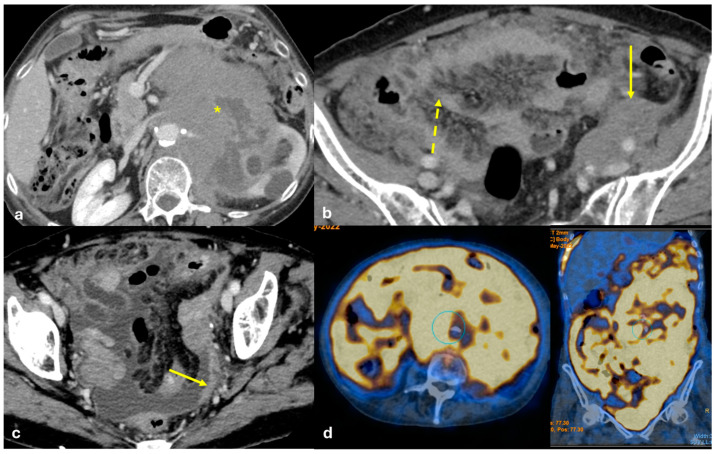
Peritoneal lymphomatosis (large B-cell lymphoma). A middle-aged woman with large B-cell lymphoma involving the left kidney, the pancreas, and the retroperitoneal space ((**a**), asterisk) with peritoneal lymphomatosis. CECT on the axial plane shows omental caking ((**b**), dashed arrow), solid tissue along the left iliac vessels ((**b**), arrow), and peritoneal seeding along the peritoneum which covers the pelvic wall ((**c**), arrow). (FDG) PET-CT shows diffuse FDG uptake of the lymphoma and peritoneal diffusion sites (**d**).

Omental caking (fine nodular soft tissue studding or large confluent soft tissue masses within the omentum) with bulky homogeneous masses is the pattern most commonly found in peritoneal lymphomatosis (Figure 8).

Other findings are small nodules in the omentum together with fine infiltration of the surrounding fat or the stellate appearance of the mesentery resulting from an infiltrating process and causing the fixation of the small bowel loops. Ascites may be present, typically being exudative with high attenuation due to the increased proteinaceous content, and usually of a small to medium volume.

Other findings may include peritoneal enhancement and thickening.

Patterns of peritoneal and mesenteric involvement in lymphoma are not specific and overlap with other causes of peritoneal disease spread, such as carcinomatosis or sarcomatosis. Findings suggestive of peritoneal lymphomatosis include the presence of enlarged lymph nodes, even in lymph nodes distant from the primary mass in association with the mesenteric disease; omental caking with homogeneous bulky masses rather than a nodular pattern; and a diffuse distribution of enlarged lymph nodes [32,33,34].

#### 3.1.6. Mesenteric Metastases from Ileal Neuroendocrine (Carcinoid) Tumors

Primary ileal carcinoid tumors originate from enterochromaffin cells located in the submucosal layer. They can be solitary or multifocal and occur in patients aged 50–70 years of age. In 10% of patients, they can cause carcinoid syndrome with classic symptoms including flushing, sweating, and watery diarrhea.

**Figure 9:**
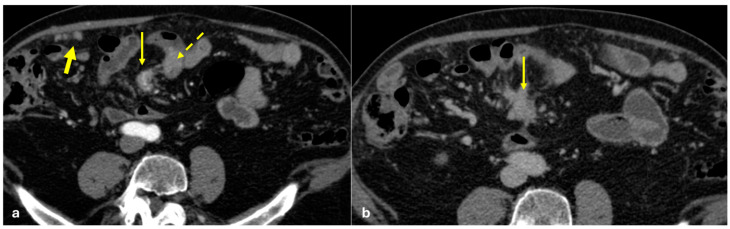
Peritoneal metastasis from ileal carcinoid. A middle-aged man with abdominal pain and episodes of subocclusion. CECT is made and shows a solid large mesenteric mass with a stellate appearance ((**a**,**b**), arrow). Focal vascularized thickening of the adjacent small bowel wall is visible and there is suspicion of a primary neuroendocrine tumor ((**a**), dashed arrow). Small nodules are visible along the large omentum and are indicative of peritoneal seeding ((**a**), large arrow).

CT has a sensitivity of 60%, which increases with the addition of early arterial phase imaging. Tumors appear as submucosal plaque-like or polypoid masses or as nodular areas of wall thickening with a crescent or C shape. They can lead to intestinal intussusception or obstruction. Fibrosis secondary to neoplastic infiltration of the bowel wall and adjacent mesentery are typical. Mesenteric metastases are frequent, occurring in nearly 50% of tumors ≤ 1 cm and 33% of tumors ≤ 0.5 cm, which present with nodal disease. Mesenteric nodules, although small, can cause progressive thickening, luminal stenosis, or occlusion of the mesenteric vessels due to vasoactive substances leading to venous obstruction of the draining mesenteric veins (Figure 9). Vascular sclerosis, together with the local effects of serotonin in the mesentery, lead to retraction, spiculation, and eventual fibrosis, resulting in a large mesenteric mass with a surrounding desmoplastic reaction, known as the classic ‘sunburst appearance’ on imaging [35].

Furthermore, 70% of lesions may exhibit stippled, coarse, or diffuse calcification. MRI has a limited role in identifying the primary tumor and mesenteric nodes, whereas it has higher sensitivity in detecting liver metastases. Since carcinoid tumors express subtype 2 and 5 somatostatin receptors, nuclear medicine has a pivotal role in disease characterization and staging, especially with 68Ga-DOTA-PET, which has over 94% sensitivity and 92% specificity.

### 3.2. Neoplastic Benign

#### Mesenteric Pseudocyst

Mesenteric pseudocysts are rare cystic abdominal masses of unknown origin with a prevalence of 1 in 100,000/250,000 hospital admissions. They can originate from any area of the mesentery or surrounding tissues, with 50–80% occurring in the small bowel mesentery, 15–30% in the large bowel mesentery, and 7–20% in the retroperitoneum [36]. They are not connected to retroperitoneal organs.

The wall of the cyst is composed of fibrous tissue without an epithelial lining [37].

In 3% of cases, mesenteric pseudocysts can be malignant, most commonly presenting as sarcoma or adenocarcinoma. Radiological features that suggest malignancy include a larger size, the presence of solid components, and rapid growth. Due to the lack of definitive malignant features and the potential for malignant transformation, complete surgical resection of the lesion is considered the treatment of choice [38].

**Figure 10:**
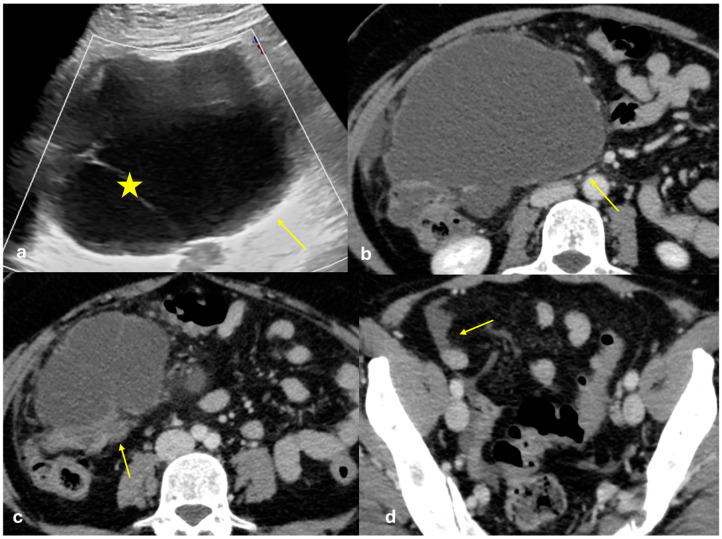
Mesenteric pseudocyst. A middle-aged woman with abdominal pain and a palpable mass in the right hypocondrium. US reveals a large cystic anechoic mass of unknown origin located next to the liver hilum with a thin nonvascularized septum ((**a**), arrow; star on the septum). CE-CT shows a large cystic mass located anteriorly to the ascending colon ((**b**,**c**): arrows). A small amount of fluid which may be secondary to cyst rupture is visible in the pelvis ((**d**): arrow).

Mesenteric pseudocysts are often asymptomatic. When symptoms are present, they are typically related to the size or location of the cyst and to complications such as infection, bleeding, or rupture [39]. Jejunal pseudocysts may present as a palpable mass that can disappear over time or change location due to the mobility of the jejunal loops (Figure 10).

US, CT, and MRI are all valuable imaging techniques for diagnosing mesenteric pseudocysts. On US, distinctive features may be seen, such as the presence of septa within the pseudocyst, a thick-walled cyst merging with the muscle layer of the bowel in cases of enteric duplication, or internal echoes suggestive of mucus or infectious cysts.

### 3.3. Inflammatory Diseases

#### 3.3.1. Epiploic Appendagitis

Epiploic appendagitis is a rare cause of acute abdominal pain resembling appendicitis and diverticulitis, but it does not require surgical intervention [40]. It results from self-limiting inflammation or ischemic damage to the epiploic appendages, or it can be secondary to inflammatory conditions of the adjacent abdominal organs [41,42].

Epiploic appendages are pedunculated adipose protrusions extending from the serosal surface of the large intestine into the peritoneal cavity and extending from the cecum to the rectosigmoid junction. They have a peculiar and limited vascular supply: the arterial supply comes from one or two small feeding arteries originating from the vasa recta longa of the colon, while the venous drainage relies on a tortuous vein passing through a narrow pedicle. Due to these anatomical features and the appendages’ great mobility, the pedicle can spontaneously twist, leading to ischemia or hemorrhage.

Unless they are inflamed or outlined by ascites, epiploic appendages are not visible on imaging because they have the same density as the surrounding fat [43].

**Figure 11:**
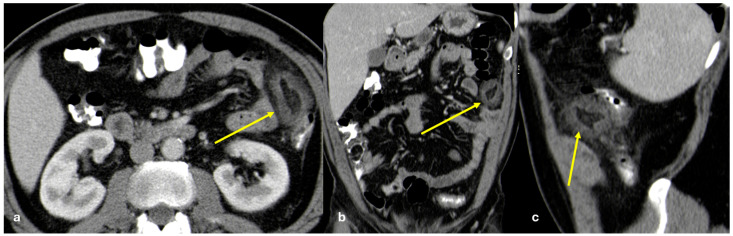
Epiploic appendagitis. A middle-aged man with abdominal pain mainly located on the left upper quadrant. Portal venous phase CT shows a fat-density, round, and small lesion surrounded by a high-attenuation rim (hyperattenuating ring sign) abutting anteriorly the descending colon ((**a**): axial, (**b**): coronal, and (**c**): sagittal images; arrows).

US is often the first imaging modality in cases of acute abdominal pain. It may show, at the point of maximum tenderness, a hyperechoic lesion surrounded by a hypoechoic rim. CT is the gold standard for diagnosing epiploic appendagitis. CT typically reveals a fat-density, round, and small lesion abutting the adjacent anterior large bowel wall, surrounded by a high-attenuation rim, referred to as the “hyperattenuating ring sign” (Figure 11). Thickening of the adjacent colonic wall is usually minimal and disproportionate to the severity of the adjacent mesenteric inflammation. In 30–78% of cases, a hyperattenuating central focus may be visible, indicative of central venous pedicle thrombosis, which is known as the “central dot sign.” Over time, infarcted epiploic appendages can evolve into a fibrotic or calcified nodule, often exhibiting a “popcorn” or “eggshell” calcification pattern [43].

#### 3.3.2. Perigastric Appendagitis

**Figure 12:**
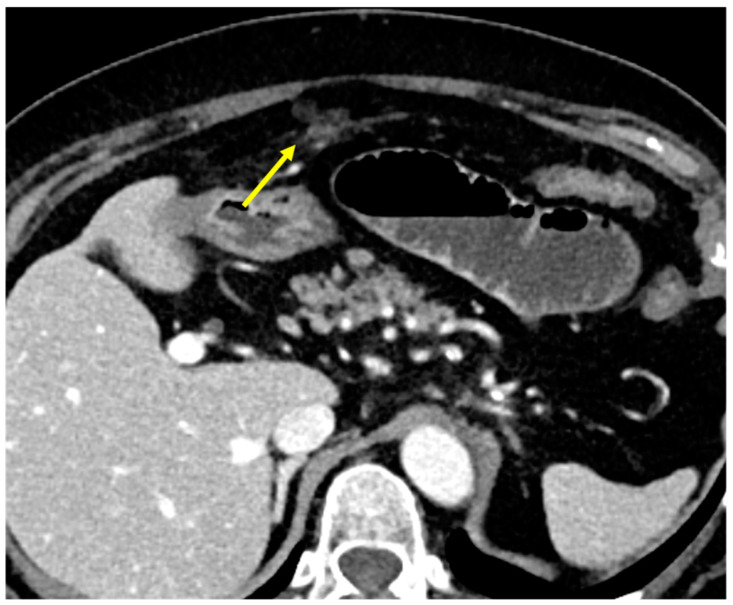
Perigastric appendagitis. A middle-aged woman with mild abdominal pain. At CT, a heterogeneous, fat-density, ovoidal lesion with mild surrounding fat stranding along the course of the gastrohepatic ligament is visible (arrow).

Perigastric appendagitis is an uncommon cause of acute abdominal pain. It results from the spontaneous twisting of an appendage attached to a perigastric ligament, leading to vascular insufficiency and infarction. This process causes focal peritonitis. The involved adipose tissue may undergo necrosis, saponification, and calcification. CT imaging typically reveals a heterogeneous, fat-density, ovoid lesion with mild surrounding fat stranding along the course of the gastrohepatic (anterior to the stomach), gastrosplenic (posterior to the stomach), and falciform (anterior to the liver) ligaments (Figure 12) [43].

#### 3.3.3. Mesenteric Panniculitis

Mesenteric panniculitis is a rare form of inflammation primarily affecting the mesenteric adipose tissue. Its etiology remains unclear, though it has been associated with cancer, abdominal trauma, previous surgeries, autoimmune diseases, and obesity. The condition is often asymptomatic and is typically discovered incidentally. When symptomatic, it manifests with nonspecific clinical signs. The most common symptom is abdominal pain, followed by nausea, vomiting, diarrhea, constipation, fever, weight loss, and chylous ascites [44].

There are two primary forms of mesenteric panniculitis: the classical form, which is more common, characterized by inflammation, necrosis, and degeneration of mesenteric fat, and the retractile form, which is mainly characterized by fibrosis of the mesentery with retraction of the surrounding structures. Some authors describe an initial pathological stage called lipodystrophic panniculitis, where mesenteric fat undergoes diffuse degeneration [45].

**Figure 13:**
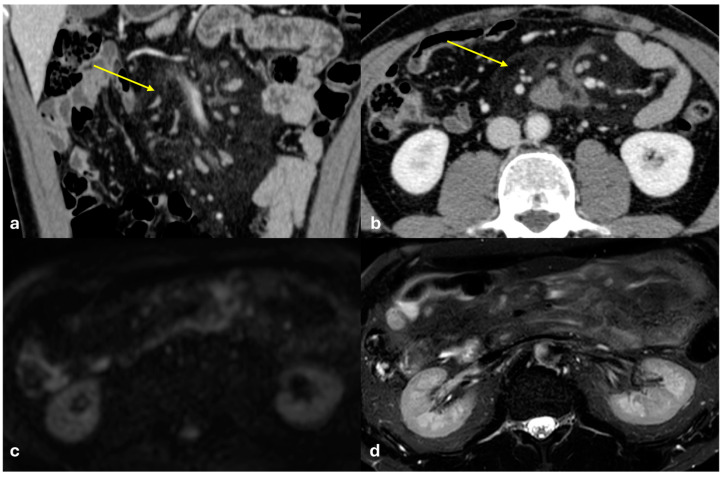
Mesenteric panniculitis. A middle-aged man, asymptomatic. CECT, made for blunt abdominal trauma, reveals hypertrophy of the mesenteric adipose tissue with soft tissue infiltration, a ring of normal fat surrounding the vessels and lymph nodes (fat ring sign) and a pseudocapsule ((**a**): coronal and (**b**): axial, arrows). MRI is made for further investigation: no significant restriction of diffusion is found (**c**) and mild hyperintensity is found on the T2-weighted image (**d**).

The diagnosis is radiological, with CT and MRI being the most accurate imaging techniques. Imaging findings typically reveal a heterogeneous, solitary mass located in the mesentery, which displaces the small intestine and surrounding structures. The two main radiological signs of mesenteric panniculitis are the “fat ring sign” and the “pseudocapsule sign” (Figure 13). The fat ring sign consists of a ring of normal fat surrounding vessels and lymph nodes; the pseudocapsule sign refers to a thin fibrotic rim, usually less than 3 mm in thickness, surrounding the mass. These signs are found in 70–92% and 50–60% of patients, respectively [46]. The mesenteric vessels can appear dilated and venous thrombosis has been described in some cases. On MRI, the classic inflammatory form is typically hypointense on T1-weighted images and hyperintense on T2-weighted sequences, whereas in the retractile form, the lesion appears hypointense on both T1- and T2-weighted sequences with a hypointense pseudocapsule, and enhancement is typically seen in the delayed phase. No significant diffusion restriction is observed on DWI [46].

#### 3.3.4. Subperitoneal Spread of Necrotizing Pancreatitis

Acute pancreatitis is an acute inflammation of the pancreas. Based on the extent of glandular involvement, it is classified as either interstitial edematous or necrotizing pancreatitis. Although acute pancreatitis can have multiple causes, the underlying mechanism is the activation of pancreatic enzymes within the gland leading to inflammation of the pancreatic tissue, disruption of small pancreatic ducts, and leakage of pancreatic secretions [47]. Since the pancreas lacks a capsule, pancreatic secretions can easily reach the surrounding tissues via the “subperitoneal space”, which connects the intraperitoneal organs with each other and with extraperitoneal tissues [2,48].

**Figure 14:**
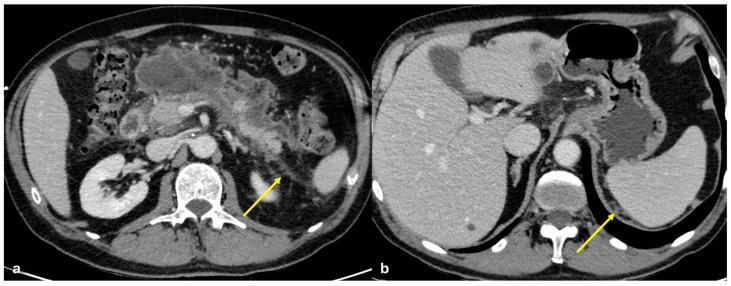
Subperitoneal spread of necrotizing pancreatitis. A middle-aged man with acute necrotizing pancreatitis. CECT made at follow-up after 7 days from the onset of the abdominal symptoms. Large necrotic collections are visible involving the pancreatic gland and the peripancreatic tissue (**a**). Small micro-nodular seeding of necrosis is visible along the posterior parietal peritoneum ((**a**,**b**), arrows), typical of pancreatitis and secondary to subperitoneal spread.

This space serves as a potential pathway for the spread of inflammatory or malignant diseases, such as pancreatitis (Figure 14) and pancreatic carcinoma.

In inflammatory conditions originating from the pancreas, fluid collections can spread along these pathways, especially anteriorly to the liver through the gastrohepatic or hepatoduodenal ligaments, or to the left through the gastrosplenic ligament.

#### 3.3.5. Pelvic Inflammatory Disease

Pelvic inflammatory disease (PID) encompasses a wide range of different infective/inflammatory disorders involving female internal genitalia and extending, through the salpinges, to the pelvic peritoneum with the subsequent development of peritonitis and abscesses. PID typically affects sexually active young females and is mostly caused by infections from Chlamydia trachomatis and Neisseria gonorrhoeae [49]. Patients with PID often report a quite long history of progressively worsening pelvic pain associated with vaginal discharge and fever. In advanced cases, the disease may extend to the upper abdomen determining perihepatitis, the so-called Fitz–Hugh–Curtis syndrome [50,51].

**Figure 15:**
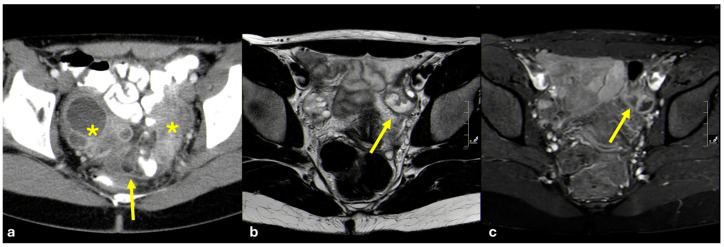
Pelvic inflammatory disease (PID). Two young women with a long history of pelvic pain and purulent vaginal discharge. In the first case, portal venous phase abdominal CT is performed (**a**) and shows the increased attenuation of the pelvic fat, peritoneal fluid with peritoneal enhancement (arrow), and complex solid-cystic-enhancing masses in both adnexal regions (asterisks); Chlamydia trachomatis is detected as the causal agent. In the second case, pelvic MRI is performed. On T2-weighted axial images (**b**), left tubal distention with thickened walls (arrow) is appreciable. On contrast-enhanced fat-saturated T1-weighted axial images (**c**), increased enhancement of the peritoneal spaces as well as of the left salpinx (arrow) is evident. The etiology in this case is polymicrobial.

Given the symptoms, patients usually undergo a gynecological evaluation with transvaginal and transabdominal ultrasound as the first-line imaging modality [52]. Ultrasound findings are non-specific and include increased pelvic fat echogenicity, dilated fallopian tubes with thickened walls, enlarged ovaries, and pelvic collections; increased endometrial vascularity as a consequence of endometritis is often observed [53]. CT and MRI are often requested to confirm the diagnosis and to assess disease extension, with the second modality being more accurate [54,55,56]. On CT, an increase in pelvic fat attenuation is almost always present, as well as a thickened parietal peritoneum and capsulated fluid collections. The adnexa and fallopian tubes typically appear as masses with a hypoattenuating core and peripheral enhancement (Figure 15a) [57]. Enlarged reactive lymph nodes are almost always present. MRI can better depict the different anatomical structures involved in inflammation. There is an overall increase in the T2 signal of pelvic structures, better depictable on fat-saturated T2-weighted images and with the diffuse enhancement of peritoneal layers (Figure 15b,c). High b-value diffusion-weighted images enable pelvic abscesses and the presence of pyosalpinx to be clearly depicted [58,59].

#### 3.3.6. Peritoneal Tuberculosis

Tuberculosis remains endemic in many developing countries and has also shown a resurgence in developed nations, primarily due to migratory flows [60]. Tuberculous peritonitis is one of the extra-thoracic manifestations of tuberculosis and is more common in patients with cirrhosis, HIV infection, and those undergoing peritoneal dialysis [61,62,63]. Patients with tuberculous peritonitis typically present with abdominal pain, ascites, and fever. Serum CA 125 values are often elevated [64]. The peritoneal effusion is exudative and shows an increased leukocyte count with lymphocytic prevalence; mycobacteria may be detected in the peritoneal fluid after centrifugation [65].

**Figure 16:**
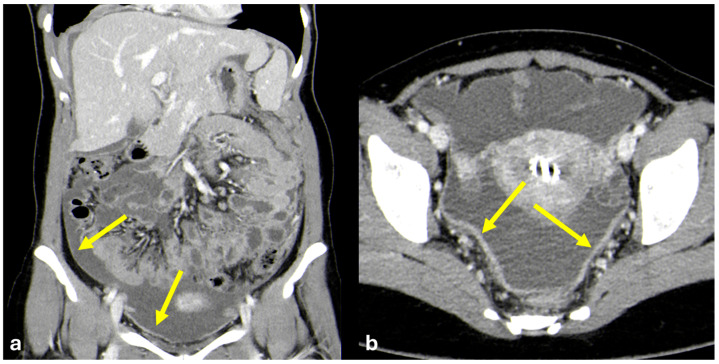
Tubercular peritonitis. A young woman with progressive abdominal pain and an increased abdominal circumference. Portal venous phase abdominal CT is performed due to the suspicion of oncological disease and shows abundant peritoneal fluid associated with diffuse and smooth thickening of the parietal peritoneum ((**a**): coronal and (**b**): axial, arrows) with no focal thickening or nodules.

**Figure 17:**
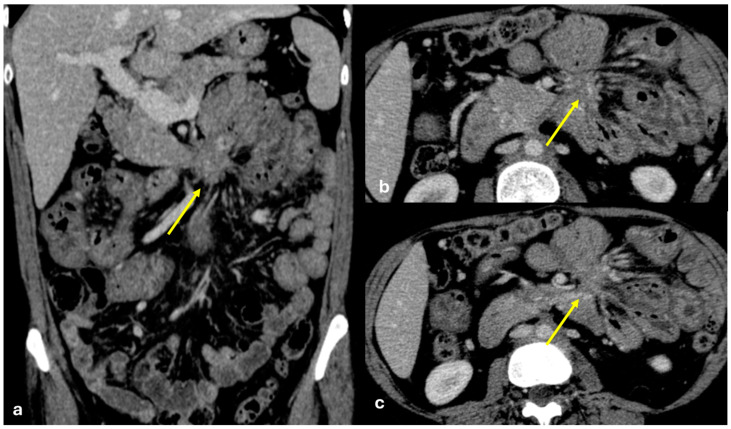
Retractile mesenteritis as an outcome of atypical micobatteriosis. A middle-aged woman with an old and long history of atypical micobatteriosis. CECT during follow-up shows a spiculated mesenteric mass with the same calcified foci ((**a**): coronal, (**b**,**c**): axial plane; arrows).

Ultrasound is often performed as a first-line imaging modality and can depict ascites and peritoneal thickening; however, these findings are non-specific. CT is usually performed to characterize these anomalies. The CT features of tubercular peritonitis may overlap those of peritoneal carcinomatosis [66,67] and include massive ascites, diffuse peritoneal thickening, and lymphadenopathy; however, the presence of smooth and diffuse peritoneal thickening, with no focal nodules, is strongly associated with peritoneal tuberculosis and, in an adequate clinical setting, enables further laboratory analyses to be correctly guided to confirm the diagnosis (Figure 16). Other features that can be found, such as omentum rim/line, lymph node necrosis or calcification, and mesenteric macro-nodules, have good specificity but limited sensitivity [68,69]. After many years, it can evolve in retractile mesenteritis (Figure 17). MRI does not offer significant advantages in diagnostic accuracy over CT [70,71].

### 3.4. Traumatic Disease

#### 3.4.1. Post-Traumatic Mesenteric Injuries

Mesenteric injuries are an uncommon cause of hemoperitoneum after blunt abdominal trauma. The lack of specific imaging features and symptoms can lead to delayed diagnosis, resulting in higher morbidity and mortality [72].

During abdominal trauma, shear and tangential forces from traction and counter-traction can cause the stretching and subsequent rupture of the mesentery, leading to mesenteric laceration. As a result, this can lead to the rupture of vessels supplying the small bowel, causing reduced vascular supply and subsequent segmental ischemia. In some cases, surgical resection may be necessary.

**Figure 18:**
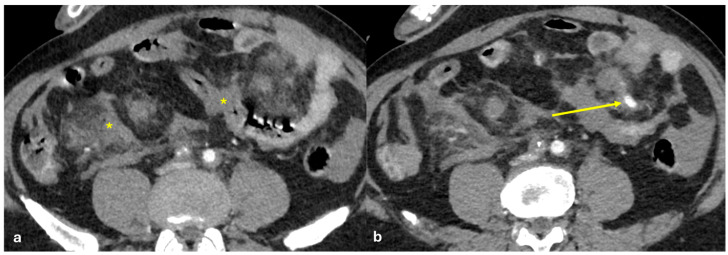
Post-traumatic mesenteric injuries. Total-body CECT made for a middle-aged man after a car accident. CECT reveals mesenteric blood infiltration ((**a**), asterisks) with active contrast leaking during the post-contrast arterial phase ((**b**), arrow).

On CT, suggestive signs of mesenteric injury include mesenteric infiltration (haziness and fat stranding), mesenteric hematoma, and bowel wall thickening. Definite signs include active contrast extravasation, free fluid within the mesentery, irregular mesenteric vessels with abrupt termination, and contrast accumulation on multiphase images (Figure 18).

#### 3.4.2. Hemoperitoneum

Hemoperitoneum is defined by the presence of blood within the peritoneal cavity. The possible etiologies are numerous and include traumatic abdominal viscera injuries, aneurysm rupture, traumatic vascular injuries, iatrogenic vascular lesions, ovarian cyst rupture, ectopic pregnancies, neoplastic bleeding, and spontaneous bleeding (mostly in anticoagulated patients) [73,74,75,76,77,78,79,80,81]. Therefore, patient history is crucial in narrowing the spectrum of possible etiologies. Patients with hemoperitoneum typically present with acute abdominal pain, low hemoglobin levels, and signs of hemodynamic shock.

Ultrasound is often performed as a first-line imaging modality in hemodynamically stable patients and shows free abdominal fluid in the peritoneal recesses. Hemoperitoneum does not have a specific appearance at ultrasound, and in the acute phase, it may appear iso-, hypo-, or hyperechoic [82,83]. Therefore, any free fluid detected in patients with major abdominal trauma and/or acute anemia must be considered to be hemoperitoneum until proven otherwise. Conversely, fluid–fluid levels and echogenic clots are usually observed in subacute and chronic stages.

**Figure 19:**
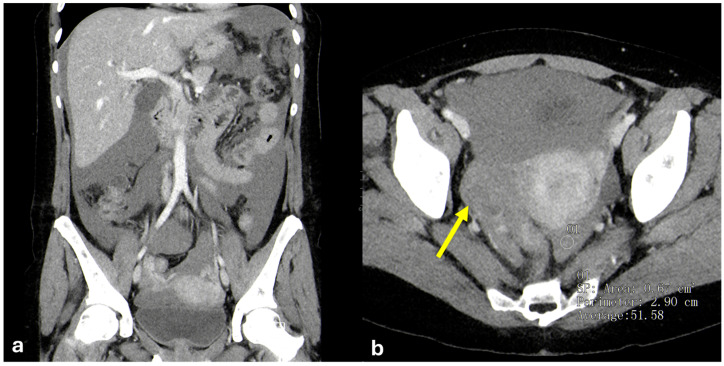
Hemoperitoneum. A young woman with acute subacute abdominal pain, anemisation, and no history of trauma. Free fluid was detected at ultrasound and portal venous phase abdominal CT was requested. Coronal CT reconstruction (**a**) shows a diffuse peritoneal effusion. On axial reconstruction (**b**), the peritoneal fluid shows high attenuation values in the dependent portions (52 HU). Moreover, the right ovary appears increased in size (arrow). The patient underwent explorative laparotomy, and a ruptured luteal cyst in the right adnexa was detected as the cause of the hemoperitoneum.

CT is the gold standard for suspected hemoperitoneum. Indeed, by measuring free fluid’s attenuation, serous effusions can be differentiated from hematic ones. In the acute phase, hemoperitoneum has attenuation values above 30 HU (typically 30–50 HU), which are typically higher in the dependent portions due to the sedimentation of red blood cells and clot formation (Figure 19). In the subacute–chronic phases, hemoperitoneum’s attenuation progressively reduces, becoming like that of serous effusions. Moreover, CT often identifies the source of bleeding [84,85].

MRI is not used in the acute setting due to its long acquisition times. Fluid–fluid levels with the “hematocrit sign”, i.e., T1 hyperintensity and T2 hypointensity in the dependent portions, are typically observed in the chronic stage.

#### 3.4.3. Splenosis

Splenosis is an acquired condition characterized by the ectopic auto-implantation of splenic tissue following trauma or surgery. Displaced splenic fragments can induce neoangiogenesis and become functionally independent, growing over time [86]. Typically, splenic implants are numerous, primarily located on serosal peritoneal surfaces, and asymptomatic, often detected incidentally during imaging studies [87]. However, they may become symptomatic, causing intestinal occlusion or abdominal bleeding [88]. According to their location, displaced splenic fragments may mimic different pathologies including peritoneal carcinomatosis, pancreatic masses, and liver lesions.

**Figure 20:**
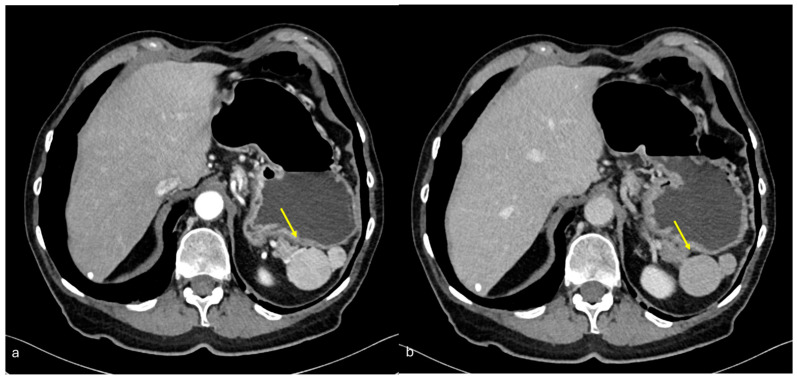
Splenosis. A middle-aged man with previous surgical intervention of a left emicolectomy and splenectomy. At CECT, rounded solid nodules (arrows) are seen in the left hypochondrium, with inhomogeneous enhancement in the arterial phase (**a**) and homogeneous enhancement in the portal phase (**b**).

Splenic implants appear as well-defined soft tissue masses, most commonly found in the upper left abdominal quadrant [89]. On both CT and MRI, the splenic fragments show attenuation/signal intensity like that of normal splenic tissue. However, in the absence of a native spleen for comparison, a definite diagnosis may be challenging (Figure 20). Nuclear scintigraphy using heat-damaged red blood cells tagged with technetium-99 enables a definite diagnosis by showing the high uptake in ectopic splenic tissue [90,91].

## 4. Conclusions

The peritoneum is composed of compartments and reflections that significantly influence the development and dissemination of disease. Recognizing normal anatomical structures is essential for distinguishing pathological alterations, such as abnormal fluid accumulations or tumor implants. Various pathologies—malignant, infectious, or inflammatory—tend to follow specific dissemination pathways within the peritoneal cavity. A thorough understanding of the peritoneal anatomy is therefore crucial for the accurate interpretation of CT and MRI findings. Familiarity with typical sites involved in disease helps prevent misdiagnosis and provides critical guidance for clinical decision-making. In summary, mastery of peritoneal anatomy and its mechanisms of disease spread enhances diagnostic accuracy, supports differential diagnosis, and ultimately contributes to improved patient management. This foundational knowledge enables radiologists to approach imaging systematically and to offer meaningful, clinically relevant insights.

## Data Availability

Not applicable.
